# Immune Parameters to Consider When Choosing T-Cell Receptors for Therapy

**DOI:** 10.3389/fimmu.2013.00229

**Published:** 2013-08-05

**Authors:** Scott R. Burrows, John J. Miles

**Affiliations:** ^1^Human Immunity Laboratory and Cellular Immunology Laboratory, Queensland Institute of Medical Research, Brisbane, QLD, Australia; ^2^School of Medicine, The University of Queensland, Brisbane, QLD, Australia; ^3^Institute of Infection and Immunity, Cardiff University School of Medicine, Cardiff, Wales, UK

**Keywords:** T-cell epitope, T-cell receptor, T-cell engineering

## Abstract

T-cell receptor (TCR) therapy has arrived as a realistic treatment option for many human diseases. TCR gene therapy allows for the mass redirection of T-cells against a defined antigen while high affinity TCR engineering allows for the creation of a new class of soluble drugs. However, deciding which TCR blueprint to take forward for gene therapy or engineering is difficult. More than one quintillion TCR combinations can be generated by somatic recombination and we are only now beginning to appreciate that not all are functionally equal. TCRs can exhibit high or low degrees of HLA-restricted cross-reactivity and alloreact against one or a combination of HLA alleles. Identifying TCR candidates with high specificity and minimal cross-reactivity/alloreactivity footprints before engineering is obviously highly desirable. Here we will summarize what we currently know about TCR biology with regard to immunoengineering.

## Background

The αβ T-cell receptor (TCR) is one of the most variable proteins known to science (1) with the human V(D)J recombination system capable of generating hundreds of trillion of unique αβ TCR molecules (2). This incredibly vast receptor reserve is our immune systems’ core defense against the torrent of hypervariable microorganisms and pathogenic challenges encountered over the course of life. During thymopoiesis, the TCR recombination machinery uses “cut-and-paste” transposition to incise and rearrange 174 variable (TRAV and TRBV), diversity (TRBD), joining (TRAJ and TRBJ), and constant (TRAC and TRBC) TCR gene segments on chromosomes 7 (TRB loci) and 14 (TRA loci) into around seven-and-a-half million distinctive gene combinations (2). This chromosomal recombination process generates only around 10% of total TCR diversity with the remaining 90% of diversity generated through further exonuclease activity and the addition of random, non-template-dependent nucleotides (N-nucleotides) across the V(D)J junction by the enzyme terminal deoxynucleotidyl transferase (TdT) (3). The collective sum of this recombination event is a theoretical 10^15^–10^20^ structurally unique αβ TCR molecules (1, 4, 5). Due to size constraints (2), the human immune system only houses an infinitely small slice of the full repertoire. In an adult human, this equates to 10^12^ T-cells (6, 7) bearing around 2.5 × 10^6^ unique αβ TCR structures (6), with the upper bounds comprising 10^8^–10^11^ unique αβ TCR structures per individual (6, 7).

The αβ TCR is a glycosylated, membrane-integral surface protein comprising one α-chain and one β-chain (2). The two chains fold and fuse via cysteine–cysteine disulfide linkers to produce a single, functional heterodimeric receptor (8, 9). The outward facing and solvent-exposed edge of the heterodimer bears six highly flexible complementarity determining region (CDR) loops. The CDR1 and CDR2 loops are encoded by the germline TRAV and TRBV genes and generally function to fix the TCR to the major histocompatibility complex (MHC) platform. Conversely, the CDR3 loops are encoded by the somatically hypervariable V(D)J junction and classically function to engage the peptide (p) cradled in the MHC groove (8, 9), although variations on CDR binding geometry have been noted (9).

A TCR engages its cognate pMHC as a single, composite ligand, and docks in an approximately diagonal fashion that slightly varies in pivot and tilt from complex to complex (9, 10). One steady constant of TCR/pMHC engagement is that the CDR3α loop is positioned toward the direction of the peptide N-terminus and the CDR3β loop is positioned toward the direction of the peptide C-terminus and variation in this geometry has not been seen to date (9, 10), however extreme terminal focusing has been recently observed (11). The TCR/pMHC docking process can be very fluid and conformational changes to the TCR, peptide, and MHC have all been observed suggesting that both interfaces often adjust to each other to find a compatible binding solution (9). Biophysical data show that TCR binding is stratified based on function. TCRs that engage pMHC class-I (pMHC-I) targets bind strongly with a mean affinity three time stronger than TCRs that engage pMHC class-II (pMHC-II) targets (8). Likewise, TCRs are further stratified based on whether the antigen target is of self or foreign origin, with foreign-reactive TCRs binding cognate pMHC with a mean affinity 10 times stronger than TCRs that bind self pMHC (8).

In spite of the large number of TCR receptor “options” available in the naive repertoire, T-cell repertoires deployed against pMHC antigens often exhibit ordered and predictable TCR gene architecture [reviewed (2, 12)]. This phenomenon, termed TCR bias, can result in residue-identical memory clonotypes being found across multiple individuals sharing a common MHC allele. The mechanisms behind the appearance of these “public” T-cell responses are still being determined (2) but is thought to involve both biases in recombination during thymopoiesis (13) and some optimal, structural-based, filtering event during antigen-driven selection (14–20). For this filtering event, it appears the peptide is the determining factor during repertoire formation (21) with TCR repertoire assembly not dependent on antigen source, presenting MHC allele or immunodominance hierarchy. Once a memory T-cell repertoire is established, it appears to remain relatively consistent both in terms of clonotype stability and clonotype frequency over decades of life (22, 23).

Adoptive cell therapy (ACT) using antigen-specific T-cells has proven to be a remarkably effective experimental treatment option for Epstein–Barr virus malignancies (24), cytomegalovirus infection (25), and melanoma (26). Given these promising results, many groups have turned toward TCR gene transfer as a simpler, faster, and more homogeneous technique for generating ACTs. Here, antigen-specific αβ TCR genes are delivered into recipient T-cells using a γ-retroviral vector, lentiviral vector, or transposon [reviewed (26)]. Another parallel approach for TCR therapy is to engineer high affinity mutants from natural αβ TCR “blueprints” using yeast display (27) or phage display (28). These TCR mutants can have their binding affinities amplified logarithmically to the low pM *K*_D_ range (28) while still retaining high specificity for antigen (28, 29). Affinity enhanced TCR can be used in two ways. First the mutants can be gene transferred into T-cells to increase antigen sensitivity and polyfunctionality (30). Second, the mutants can be used in soluble form to deliver therapeutic payloads to cells bearing the appropriate pMHC targets (31). Importantly, before considering a receptor for therapeutic ends, a number of parameters should be considered regarding the genetics and biology of the human TCR.

## Consideration One: Cross-Reactivity

The first parameter to considering when applying TCR therapy is the cross-reactivity profile of the candidate receptor. A theoretical proposal (32) predicted that the αβ TCR must intrinsically encode a high degree of cross-reactivity in order to provide sufficient coverage against the huge constellation of pMHC complexes that could be encountered in nature. Through the use of combinatorial peptide libraries (CPLs), that comprise almost all possible peptides of a particular length, this theory was recently tested experimentally and proven (33, 34). In the context of a single MHC, a single αβ TCR can recognize over one million different peptides as well or better than its cognate ligand (34). Whether this is the case for *all* TCRs is under active investigation. Very recent CPL data suggests that TCRs have sliding cross-reactive intensities (35) and, at least for pMHC-I-specific TCRs, an explicit preference for peptides of defined length. Thus, cross-reactivity for peptides outside a TCRs “programmed length preference” is unlikely.

Given the intrinsic cross-reactivity of TCRs, it is tempting to select for TCR that engage multiple target pMHC. Indeed, this “multiple birds with one stone” approach could dramatically boost therapeutic efficacy of a candidate TCR *in vivo*. However, caution is advised in this pursuit as it has recently been shown that multi-pMHC specific TCR can result in serious side effects (36). Here, a therapeutic TCR that recognized multiple MAGE-derived peptides resulted in neurological toxicity when administered to melanoma patients as TCR gene therapy. Off-target toxicity was thought to be due to one of the MAGE peptides being expressed in the brain. This localized expression profile was not previously known.

Another parameter to consider (on top of the large numbers of proteogenic peptides T-cells can recognize) is the issue of “transformed self.” It is known that αβ T-cells can engage proteogenic peptides containing post-translational modifications, such as phosphorylation (37), glycosylation (38), citrullination (39), and dimerization (40). Whether a given αβ TCR also cross-recognizes large numbers of modified peptides is yet to be determined. In addition to classical pMHC-I and pMHC-II targets, αβ TCR are also now known to bind a growing list of classical and non-classical MHC molecules which cradle an extraordinary diverse array of organic and inorganic compounds (41). TCR ligands can include proteogenic peptides in HLA-E (42), lipids in the cluster of differentiation 1 (CD1) molecule (41, 43), vitamin metabolites in MHC-I related (MR1) molecules (44), small molecule drugs in MHC-I (45), and the empty platform of human hemochromatosis protein (HFE) (46).

Precisely mapping the complete cross-reactive profile of a therapeutic TCR candidate across the thousands of classical and non-classical MHC alleles which present a combined universe of organic and inorganic compounds is currently possible but difficult. Basic approaches are available for assessment (Table 1). For instance, scanning a group of candidate TCRs across a CPL library can quickly rule out receptors with extensive pMHC cross-reactivity footprints. From these select receptors, blasting the raw CPL data across the human proteome may identify self peptides which could drive off-target activity *in vivo*. Candidate TCR with minimal cross-reactivity footprints as suggested by CPL scanning could then advance to *in vitro* testing on multi-cell subsets. Here, various cell types (monocytes, DCs, T-cells, B-cells, fibroblasts, epithelial cells, etc.) that express the HLA restriction allele of interest could be used as target cells to determine potential TCR cross-reactivity with self pMHC molecules. Target cells could be derived from primary sorted cells and/or cell lines.

**Table 1:** **Pre-clinical testing options for therapeutic TCR candidates**.

Parameter to consider when choosing a candidate TCR for therapy	Testing option/s
Could the candidate TCR cross-react with a peptide presented by an autologous classical and non-classical MHC molecule?	Scan the candidate TCR across different primary cell subset targets (monocytes, DCs, B-cells, T-cells) sorted from prospective patients.
	Scan the candidate TCR across PBMC and cell lines (monocytes, DCs, B-cells, T-cells, fibroblast, epithelial) from a library of HLA allele matched healthy donors.
	Scan the candidate TCR across peptide length-matched CPL to establish a metric of cross-reactivity potential.
Could the candidate TCR alloreact with a peptide presented by a mismatched MHC molecule?	Scan the candidate TCR across an extensive, fully HLA haplotyped cell line library. The cell line library should contain HLA alleles found at high frequency in the target population.
Are the germline sequences for the candidate TCR donor/patient matched?	Compare the TRAV, TRAJ, TRBV, and TRBJ sequences of the candidate TCR with patient TR loci. Polymorphisms in these genes may alter the effectiveness of the therapeutic TCR *in vivo*. Additionally, if the donor/patient TR alleles do not match, or if the patient has a key TR allele deleted, there is a possibility that a patient-derived immune response could be mobilized against the “foreign” TCR.
Could the candidate TCR steer functional phenotype of recipient T-cells when used in gene therapy?	Transduce the candidate TCR in naive T-cells *in vitro* or into mice with human immune system components. Prime the cultures with differing concentrations of cognate Ag and monitor cell fate decisions. Note temporal and final ratios in effector, memory and Tfh differentiation.
	Transduce the candidate TCR in memory T-cells *in vitro*. Prime the cultures with differing concentrations of cognate Ag and monitor if cell fate is altered when compared to phenotype pre-transduction.

## Consideration Two: Alloreactvity

As mentioned above, a significant degree of degeneracy in peptide recognition likely evolved to ensure that the TCR repertoire has the capacity to recognize the enormous variety of foreign peptides that are encountered throughout life. Furthermore, broadly reactive T-cells may aid primary and memory responses where memory T-cells for one pathogen are reactivated by a different infectious agent (47). However, limited specificity of self-MHC-restricted T-cells is also the basis of the alloresponse and its associated clinical problems.

T-cell allorecognition occurs when the immune system is presented with MHC molecules of a different allotype to that of the host. Alloreactivity becomes clinically significant in the case of solid-organ grafts or bone marrow transplants in which mismatched MHC molecules can potentially result in organ graft rejection or graft versus-host disease (GVHD). This response can be either direct, in which the T-cells mount an immune response to the foreign-pMHC, or indirect, a chronic self-MHC restricted response resulting from polymorphism in the processed antigen that can include peptides from allogeneic MHC molecules (48). It is estimated that up to 0.1–1% of T-cells are alloreactive toward a given allogeneic MHC molecule (49). However, the probability of a TCR reacting with any allogeneic MHC molecules is obviously much higher due to MHC polymorphism, and this is a potential problem for TCR therapy.

There are numerous reports of T-cell clones with dual specificity for an allo-MHC molecule and a nominal antigen complexed with self-MHC (50). The best characterized example is the response to the Epstein–Barr virus epitope FLRGRAYGL, that binds to HLA-B8, in which CTL clones were isolated that cross-reacted with one of three common alloantigens (HLA-B44, B14, or B35) (51, 52). Interestingly, the HLA-B44 alloreactive TCR [which has also been shown to alloreact with HLA-B∗5501 (53)] is a public TCR that dominates the response to this viral epitope in most HLA-B8^+^ people (54, 55). By examining the response to this viral epitope in individuals who co-expressed HLA-B8 and one of the alloantigen targets, subdominant TCRs were identified that were not alloreactive (55, 56). Such TCRs would be the obvious choice for use in TCR therapy, and this approach could be used in other systems to identify non-alloreactive TCRs for therapeutic use where the dominant receptors are alloreactive.

Many other T-cell clones have been shown to cross-react with alloantigens, and work from Frans Claas’s group has shown that up to 45% of virus-reactive T-cell clones from humans are alloreactive (50). Allo-HLA cross-reactivity was shown from T-cell clones raised against a range of viruses including cytomegalovirus, varicella-zoster virus, and influenza (50). These included both CD8^+^ and CD4^+^ clones alloreacting with MHC-I and MHC-II molecules, respectively, and surprisingly, they also included two distinct cytomegalovirus-reactive, MHC-I-restricted T-cell clones that recognized allogeneic MHC-II molecules (57).

The obvious way to manage the problem of T-cell alloreactivity in the context of TCR therapy is to perform preliminary *in vitro* screens of the TCR for cross-recognition of cell lines expressing a wide range of allo-HLA alleles. The limitation here is that it will be near impossible to screen against the huge variety of HLA molecules, given there are over 6,000 known class-I alleles and over 1,000 class-II alleles. Furthermore, alloreactive T-cells are generally also specific for one or more “self”-peptides presented by the allo-HLA molecule, and these may not be presented by cells from all tissues, or they could be derived from polymorphic gene products and are therefore not presented by all individuals or cell lines. For example, the EBV-reactive TCR described above is specific for a “self”-peptide derived from an ATP binding cassette protein ABCD3 which is presented by allo-HLA-B44 and shares only one residue with the viral peptide (58). This peptide appears to be presented at different levels in distinct tissues based on the recent observation that these T-cells recognize HLA-B44^+^ lymphoid cells but not epithelial and endothelial cells (59).

Although T-cell cross-reactivity with alloantigens has not proven to be a major problem in adoptive T-cell transfer clinical trials, it is an issue that should not be ignored in future trials of TCR therapy. Testing for cross-reactivity with one or more alloantigens is currently possible *in vitro* through target cell screening across large allo cell libraries (50, 53).

## Consideration Three: Polymorphism

As with vaccines that elicit T-cell responses against a limited number of epitopes, TCR-based therapeutic approaches need to address the important issue of polymorphism in the genes involved in antigen presentation and those encoding for the target peptide antigens. Viral antigens are particularly prone to accumulating escape mutations, and so TCRs that recognize regions of viral proteins that are critical for viral fitness and are therefore highly conserved (60) should be favored for TCR therapy. Genetic instability is also a common feature of cancer cells, often resulting in the selection of antigenic variants by T-cells which allow cancer cells to escape destruction (61). The simultaneous administration of multiple TCRs that target different epitopes should circumvent these problems to some extent. Another potential mechanism through which human genetic polymorphism could create problems is if a TCR, transferred into an unrelated recipient, cross-reacts with a polymorphic self-peptide which it had not encountered during thymic negative selection, leading to damage of healthy tissue.

HLA polymorphism is also a major consideration that restricts the potential value of individual TCRs to a limited subset of any given population. As mentioned above, a huge number of HLA alleles have now been identified and therefore TCR therapy will need to be personalized to ensure recognition of epitopes presented by relevant HLA alleles. TCRs that recognize antigenic peptides that are presented by multiple HLA alleles are also valuable candidates for TCR therapy. A degree of degeneracy in HLA-peptide binding has been demonstrated whereby multiple class-I alleles can share common sequence motifs due to homology of amino acids within the major pockets of the peptide binding cleft, and these groups of alleles are referred to as HLA supertypes. Based on HLA structural similarities and overlapping peptide binding motifs, nine major HLA supertypes have been proposed (62). Examples of TCRs that have the capacity to recognize individual peptides bound to multiple members of an HLA supertype have been described (63–66). These TCRs with promiscuous HLA restriction can often accommodate differences in the exposed HLA α-helix residues between the restricting MHC and foreign MHC antigens that present the same peptide.

As with the MHC genes, allelic sequence variation is also a feature of the TCR and this issue needs to be addressed in the context of TCR therapy. Several sequencing studies have revealed considerable polymorphism within the TRAV and TRBV gene segments (67, 68). In one study, the TCR loci from 40 individuals across four ethnic groups were fully sequenced, and more than 550 SNPs were found, with many being situated in coding/regulatory regions of functional TCR genes and several causing null and non-functional mutations. On average, the coding region of each TCR variable gene contained two SNPs, with many more found in the 5′, 3′ and intronic sequences of these segments. A total of 51 SNPs in the TRA locus and 72 SNPs in the TRB locus were found to result in amino acid changes (67, 68).

The extensive variability within the TCR gene segments raises the interesting possibility that, unless the TCR genetics are matched between donor and recipient, some TCR gene products will be seen as foreign antigens and could elicit an immune response that limits the efficacy of transferred TCRs. Particularly strong immune responses could be expected in patients with deletions or inactivating polymorphisms that prevent expression of certain TRBV genes. There are seven frequently occurring inactivating polymorphisms in functional TRBV gene segments and a large (21.5 kb) insertion/deletion related polymorphism in the TRB locus encompassing two V gene segments (67–70). In the latter case, two functional variable β genes, *TRBV6-2*/*TRBV6-3* and *TRBV4-3*, are frequently deleted in all major ethnic groups (68, 70, 71). TCRs that are encoded by V genes that include common polymorphisms could perhaps be avoided for use in TCR therapy.

## Consideration Four: Functional Phenotype

Recent evidence suggests that different TCRs expressed by T-cell clones of the same pMHC specificity can have different effects on immune phenotype (72). When challenged with pathogen, clonotypically distinct naive T-cells were observed to give rise to differing ratios of Th1 and Tfh progeny. These alternate differentiation programs were dependent on pMHC dwell time and/or Ag density. Interestingly, this data suggests that different TCR clonotypes of the same pMHC specificity may impart differential effects on total immune function through varying effects on macrophage activity and antibody section by B-cells. An additional complexity in this area is the observation that after priming, a single naive T-cell can have multiple fates when proceeding down the cell differentiation pathway (73, 74). Thus, determining exactly which differentiation program a candidate TCR induces is an important parameter when considering a receptor for therapeutic use.

## Concluding Remark

The TCR is an extremely effective tool for targeting biological and non-biological molecules and vast opportunity exists to exploit these receptors therapeutically. However, TCRs are highly polymorphic by nature and intrinsically encode a considerable degree of differential functionality and cross-reactivity across a number of MHC and MHC-like molecules. These factors require that therapeutic TCR candidates are donor/patient matched and undergo the most comprehensive *in vitro* cross-reactivity testing we can perform with present technology. The goal of this testing should be the identification of receptor candidates with predictable cell differentiation “programs” and minimal and traceable cross-reactivity/alloreactivity footprints.

## Conflict of Interest Statement

The authors declare that the research was conducted in the absence of any commercial or financial relationships that could be construed as a potential conflict of interest.
